# Improving Peach Fruit Yield and Quality Using Foliar Application of Nano Chelated Zinc and Seaweed Extract (*Spirulina platensis*): A Multivariate Analysis Approach

**DOI:** 10.1002/fsn3.71838

**Published:** 2026-04-26

**Authors:** Ahmed Isam Dawood, Saad Abdulmajeed Waheeb, Lina saadoun Abdul majeed, Heidar Meftahizadeh

**Affiliations:** ^1^ Department of Horticulture and Landscape Architecture, College of Agriculture University of Kirkuk Kirkuk Iraq; ^2^ Department of Horticultural Science, Faculty of Agriculture & Natural Resources Ardakan University Ardakan Iran

**Keywords:** food security, horticulture, multivariate analysis, nanotechnology, nano‐zinc

## Abstract

The twenty‐seven identical peach trees were exposed to different levels of a Nano Chelated fertilizer contain 12% Zn and *Spirulina platensis* liquid extract in a factorial experiment based on RCD, in order to enhance fruit yield and quality. The maximum values of leaf area (35.9 cm^2^), SPAD (51.2 value), fruit set (28.2%), fruit firmness (26.3 lb. inch^−2^), fruit diameter (137 mm), individual fruit weight (116.3 g), flesh weight of fruit (106.7 g), seed weight (9.6 g), fruit number per tree (534.7 *n*), fruit yield per tree (62.2 kg), fruit yield (31.5‐ton ha^−1^), TSS (13.15°Brix), and vitamin C content (12.90 mg/100 mg FW) were obtained under application of *Spirulina platensis* at 2500 ppm plus Zn NPs at 6 mL L^−1^. Furthermore, the lowest fruit decay (33.33%) was observed under this combined treatment. Application of this treatment significantly improved leaf area (8.2%), SPAD (58.3%), fruit set (32.8%), fruit number (46.5%), fruit diameter (30.5%), fruit weight (41.7%), flesh weight (42.0), seed weight (37.9%), fruit yield per tree (107.5%), fruit yield ha^−1^ (107.5%), fruit firmness (62.9%), fruit decay (−44.5%), TSS (83.7%), and total phenol (6.6%^ns^) compared to control. Principal component analysis (PCA) confirmed results of means comparison and recognized 2500 ppm *Spirulina platensis* extract +6 mL L^−1^ Zn NPs as the optimal treatment to maximize yield and growth traits while maintaining low decay and high biochemical quality. Furthermore, Pearson's correlations and regression analysis revealed the strong positive relations between physical, morpho‐physiological, and yield variables. Based on the results of univariate analysis and confirmation with multivariate analysis, Foliar application of 2500 ppm seaweed in combination with 6 mL L^−1^ Zn NPs has been recommended for the improvement of fruit quantity and quality in peach trees.

## Graphical abstract

The combined application of nano‐zinc and Spirulina extract enhances peach yield, fruit firmness, and nutritional quality. An optimal treatment significantly improves fruit set (+107.5%), TSS, and vitamin C content.

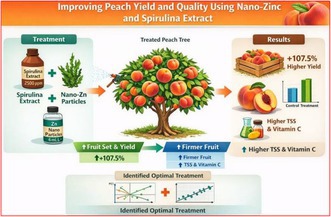

## 1 Introduction

Peach ( *Prunus persica* L. Batsch) is a vital fruit species in the Rosaceae family, a commercially important and globally recognized tree native to Southeast Asia and widely cultivated in temperate and subtropical regions (Ghahremani et al. 2023; Pérez et al. 2020; Rodríguez‐Robles et al. 2024). Peach is a nutritional and commercial summer fruit with a healthy and delicious taste (Ezzat et al. 2024; Hussain et al. 2021; Mrázová et al. 2021). Peach fruits are consumed as fresh fruit, juice and jam. This fruit is a high source of protein, carbohydrates, antioxidant compounds, fiber, vitamins (A, B and C), and vital minerals (Dinkecha and Setu 2021; Mrázová et al. 2021).

For optimum fruit production, adequate fertilization, especially micronutrient nutrition is a vital procedure, because of its effectiveness on vegetative growth, flowering process, fruit set, and fruit yield and quality (Karunakaran et al. 2025; Wang et al. 2025). Although chemical fertilizers play a vital role in agricultural production, in the long term they reduce the diversity of soil bacteria, organic matter, and soil fertility (Tadayon et al. 2025; Wang, Wu, et al. 2023). Thus, fertilization with eco‐friendly, natural fertilizers is necessary to achieve the high nutritional value of fruits and their performance (Dasgan et al. 2023; Montaño‐Herrera et al. 2022; Wang, Li, et al. 2023).

Application of PGRs and biostimulants such as Nano‐materials and biofertilizers is a promising system due to plant nutrition efficiency and sustainability, and their potential in the agri‐food sector, attracting the attention of horticultural researchers (Hussein et al. 2024; Karnwal et al. 2023; Magnabosco et al. 2023; Singh, Sharma, et al. 2024). Furthermore, the use of biostimulants increases plant yield and quality due to increased nutrition and water efficiency, while reducing the use of chemical fertilizers (Meena et al. 2025).

Seaweed extract, a nontoxic, eco‐friendly biostimulants, biostimulant, is used in sustainable agriculture (Ali et al. 2021; Kumar et al. 2024; Rabhi et al. 2025; Staykov et al. 2025). Various reports have documented the beneficial effects of seaweed bioactive substances in horticultural production (Deolu‐Ajayi et al. 2022; Lomartire and Gonçalves 2022; Pradhan et al. 2021; Zhang et al. 2022). These bioactive materials amplify the resistance of fruit trees to stress and enhance the efficiency of fertilizers (EL Boukhari et al. 2020; Gatti et al. 2025; Supriya et al. 2024). Seaweed provides a substitute for soil supplements, which heal certain adverse effects associated with fertilizers, such as soil degradation, soil acidification, and leaching of nutrients (Mahmoud et al. 2024; Benita et al. 2025; Rafi et al. 2025; Yasmeen et al. 2025). Seaweed‐derived compounds influence microbial communities, promoting beneficial interactions that improve soil structure and fertility (Rabhi et al. 2025, Akil et al. 2025).

Seaweeds contain auxins, cytokinins, gibberellins, amino acids, and macro‐ and micronutrients (Ali et al. 2022; Kumawat and Kumawat 2023; Ouala et al. 2025; Rabhi et al. 2025). Furthermore, seaweeds are rich in polysaccharides, oligosaccharides, various enzymes, vitamins, various secondary metabolites, and proteins that increase disease resistance by regulating molecular, physiological, and biochemical mechanisms in plants (Agarwal et al. 2021; El‐Beltagi et al. 2022).

Nanotechnology as an advanced eco‐friendly method upraises horticultural productivity without forfeiting fruit quality due to the special and unique characteristics of Nano‐fertilizers (NPs) (Alam et al. 2024; Suliman et al. 2025; Pagano et al. 2025; Singh, Sharma, et al. 2024; Ul Huda et al. 2025). Nano‐fertilizers (NFs), including micronutrient NPs, are low‐cost and natural–friendly products with high mobilization, higher reactivity and greater nutrient‐use efficiency in plants, resulting improves plant growth and development without toxic effects (Khan et al. 2025; Minello et al. 2025; Stojanova et al. 2025).

Zinc oxide nanoparticle (ZnO‐NP), a metal oxide nanomaterial, is valuable and versatile inorganic material with physical and chemical characteristics (Zhou et al. 2023). ZnO NPs perform better compared to Zn orthodox fertilizers owing to their enhanced uptake and translocation in plants, improving crop growth compared to other Zn sources (Adil et al. 2022; Mi et al. 2023; Singh, Saffeullah, et al. 2024). ZnO NPs have resulted in the development of an innovative line for advancing plant growth and efficiency through targeted transfer and a slow‐release mechanism, regulating phytohormone levels, enhancing nutrient use efficiency, root morphology, and enzyme activity (Hanif et al. 2024). Application of 3 g L^−1^ nanoparticles containing iron 8%, zinc 1.5%, copper 0.5%, manganese 1.5%, boron 0.5%, and molybdenum 0.5% significantly enhanced plant height, stem diameter, leaf number, leaf area, mineral concentration, chlorophyll content, and carbohydrate levels in orange leaves (Mittal et al. 2020; Hamzah and Ibrahim 2024).

In horticulture fertilization, ZnO‐NPs supplementation is an effective practice to improve growth, yield, and fruit quality (Ahmad and Anjum 2023; Manzoor et al. 2025). For examples: Supplementation of nano‐Zn before flowering led to greater plant resilience to floral malformation disease and improved fruit quality in pomegranate and mango (Anjum et al. 2011; Zagzog and Gad 2017). The foliar sparing of 25 ppm ZnO NPs significantly increased the TSS, sugars, and anthocyanin content in berries of Table Grapes Cv. Crimson Seedless in association with decreasing acidity. Furthermore, application of 50 ppm ZnO NPs notably enhanced fruit firmness (Abou El‐Nasr et al. 2021, Anushi et al. 2023). In palm trees, foliar spraying application of 250 mg L^−1^ ZnO NPs significantly improved fruit weight, volume, total soluble solids, total and reducing sugars, total soluble proteins, and yield by 29.16%, 28.61%, 11.00%, 5.65%, 7.96%, 16.00%, and 28.76%, respectively, while reducing sucrose content by 30.02% (Mahdi 2024).

Zinc (Zn) is a mandatory trace element for plant physiological processes, such as photosynthesis, carbohydrate and protein metabolism, and growth regulation, and it is a decisive plant fertilizer for stimulating the crop yield and quality (Liu et al. 2025; Veena and Puthur 2022). Furthermore, Zn is an essential activator for several plant enzymes and is directly involved in the biosynthesis of growth hormones, resulting in improved cell division and cell elongation (Mahmoud et al. 2020). Thus, zinc deficiency causes disruption of metabolism, growth and yield restriction (Ahmed, Deng, et al. 2024; Sethi et al. 2025).

Despite the well‐documented benefits of seaweed extracts and zinc nanoparticles individually, limited information is available regarding their combined (synergistic) effects on fruit yield, quality, and post‐harvest characteristics of peach. Moreover, previous studies have rarely employed multivariate statistical approaches to comprehensively evaluate the relationships among physiological, yield, and quality traits under such treatments. Therefore, this study was designed to fill this research gap by investigating the interactive effects of *Spirulina platensis* extract and nano‐chelated zinc on peach trees. We hypothesized that the combined application of seaweed extract and nano‐zinc would synergistically enhance fruit yield and quality, while reducing post‐harvest decay compared to their individual applications. Accordingly, the main objectives of this study were: (1) to evaluate the effects of different levels of Spirulina extract and nano‐zinc on growth, yield, and fruit quality of peach; (2) to assess post‐harvest fruit decay; and (3) to analyze the relationships among measured traits using multivariate statistical methods.

## 2 Materials and Methods

**FIGURE 1:**
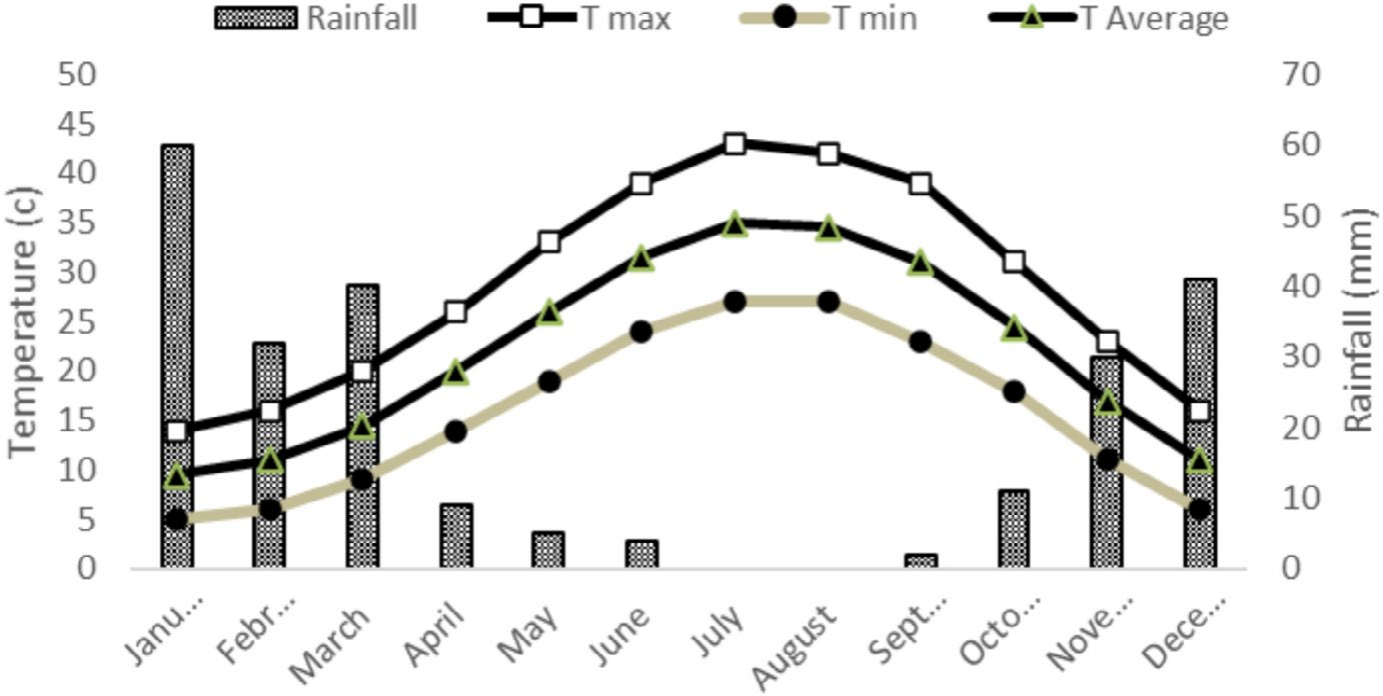
The thermo‐pluviometry trend diagram at the research orchard (Mosul, Iraq) during 2024.

The experiment was conducted in a private orchard in Dohuk Governorate, Iraq, at coordinates 36°52′04.2″N and 43°42′4.1″E in 2023. Twenty‐seven identical peach trees 7 years old and a planting spacing of 5 × 4 m were exposed to different combined treatments of *Spirulina platensis* seaweed and Nano Zinc. The specification of the orchard soil was presented in Table 1, and the climatic data in Figure 1.

**TABLE 1:** The chemical and physical properties of the orchard soil.

Acidity (%)	EC (dS.m^−1^)	O.M. (%)	N (mg L^−1^)	P (mg L^−1^)	K (mg L^−1^)	Fractions (%)			Texture
						Sand	Clay	Silt	
7.0	0.50	2.25	430	0.27	2.20	38.05	32.7	29.25	Clay Loam

The factorial experiment was conducted based on a Randomized Complete Blok Design (RCBD) with three replicates and two factors: the first factor was spraying seaweed extract (*Spirulina platensis*) at concentrations of 0, 2000, and 2500 ppm, and the second was spraying Nano Chelated Zinc at concentrations of 0, 3, and 6 mL L^−1^ at four times, with intervals of 15 days. Commercial Nano Chelated Zinc fertilizer contains 12% Zn (size below 100 nm and 90% purity) and was prepared from Khazra Company Tehran, Iran. Also, Commercial *Spirulina platensis* liquid extract (Bio‐AlgePro‐Ex) provided by PhytoBiochem, Egypt, containing a high percentage of amino acids and nitrogen, was used. Starting April 9, 2024, the trees were sprayed with *Spirulina platensis* extract and nano zinc every 15 days for 2 months. A hand spray with a capacity of 10 L was utilized until achieving complete wetness, incorporating Tween‐20 at 0.1 mL L^−1^ as solubilizers to minimize the surface tension of the water molecules.

### 2.1 Measurements of Variables

The leaf area (cm^2^) was determined using a Cl‐202 portable laser leaf area meter for thirty leaves per treatment and the average was calculated. Total chlorophyll (SPAD value) was estimated using the SPAD 502 chlorophyll measuring device in the field for thirty leaves, and the average SPAD value was calculated. The average of thirty leaves for each treatment was weighed to record the fresh weight (g), then washed with distilled water and dried in the oven at a temperature of 70°C until a constant weight was reached and weighed to record the dry weight (g). Fruit fresh weight (g) was measured for thirty fruits using the digital balance and the yield per tree (kg) and total yield per donum (kg) were calculated. Similarly, the flesh weight and seed weight (g) were measured. Fruits dry weight (%) was carried out via random samples of 100 g of fresh weight which were dried in an electric oven at 70°C till the constant weight, then the samples were weighted. Flesh weight (g) was determined by weighing the whole fruit and then subtracting the weight of the peel and seeds. Fruit size (diameter) (mm) was measured using a digital caliper. Fruit size was determined as fruit diameter (mm) using a digital caliper. Measurements were taken at the equatorial region (widest part) of each fruit to ensure consistency. A total of thirty fruits per treatment were randomly selected, and the average value was calculated for statistical analysis.

Chemical properties of fruits were evaluated as follows: the juice sample was extracted from each replicate. The total acidity was determined in accordance with AOAC (1995). Vitamin C content was measured as mg L^−1^ ascorbic acid/100 mL fresh weight by titration against 2, 6‐dichlorophenol‐indophenol (AOAC 1995). Fruit firmness (kg cm^−2^): was measured using a pressure firmness tester (Model GY‐3 UC) and calculated as Ib inch^−1^ (Equation 1).

(1) $$\text{Pound}-\text{force}\;\left(\text{lbs}\right)x\;4.448=\text{Newton}\;\left(N\right)$$

Total soluble solids (TSS % w/w), or degrees Brix (°Brix), is the percentage of sugar and other dissolved (Mostofi 2011) was estimated in the juice of the fresh fruits using a hand refractometer MA‐871, Milwaukee, Romania.

At the end of the experiment, the fruit samples 30 fruits at maturity stage uniform in size and color were taken randomly from each replicate and packed inside plastic boxes and stored under cold conditions in a smart fridge cooler. The temperature was adjusted on cold condition (4°C ± 1 with 92% ± 1 RH). The percentage of fruit decay was calculated at the end of the experiment after 40 days of storage. The decay percent was determined for thirty fruits based on five scores (no decay) = 0, light (25%) = 1, moderate = 2 (25% to 50%), and severe = 3 (more than 50%). Substantially, the decay percent was determined by Equation (2) (Eroğul et al. 2024).

(2) $$\left[\left(1\times \mathrm{FN}1\;2\times \mathrm{FN}2\;3\times \mathrm{FN}3\right)\times \frac{100}{3\times \mathrm{FN}}\right]$$

FN: the total number of fruits, FN1, FN2, and FN3 are the number of fruits showing different decay scores (Rodrıguez‐Delgado et al. 2001).

### 2.2 Statistical Analysis

Data were analyzed using a combination of univariate and multivariate statistical methods. The Two‐Way ANOVA was performed using IBM SPSS Statistics 27.0.1, Tukey's HSD test was applied following significant ANOVA results and were presented as MS ± SD. The Principal Component Analysis, Pearson's Correlations Estimation, and Linear Regression Analysis were done using R Ver. 4.5.0.

## 3 Results

The main effects of seaweed (*Spirulina platensis*) and Zn NPs were significant at the 0.01 level for SPAD value, fruit weight, fruit number, fruit yield per tree, yield ha^−1^, fruit diameter, flesh weight, seed weight, and fruit flesh weight per fruit seed weight. Furthermore, seaweed significantly (*p* ≤ 0.01) influenced the fruit set. The synergetic effect of Zn NPs and seaweed (*Spirulina platensis*) was significant (*p* ≤ 0.01) for SPAD, fruit weight, fruit number, fruit yield per tree, yield ha^−1^, fruit diameter, seed weight, flesh weight per seed weight, fruit decay, and phenol content.

**FIGURE 2:**
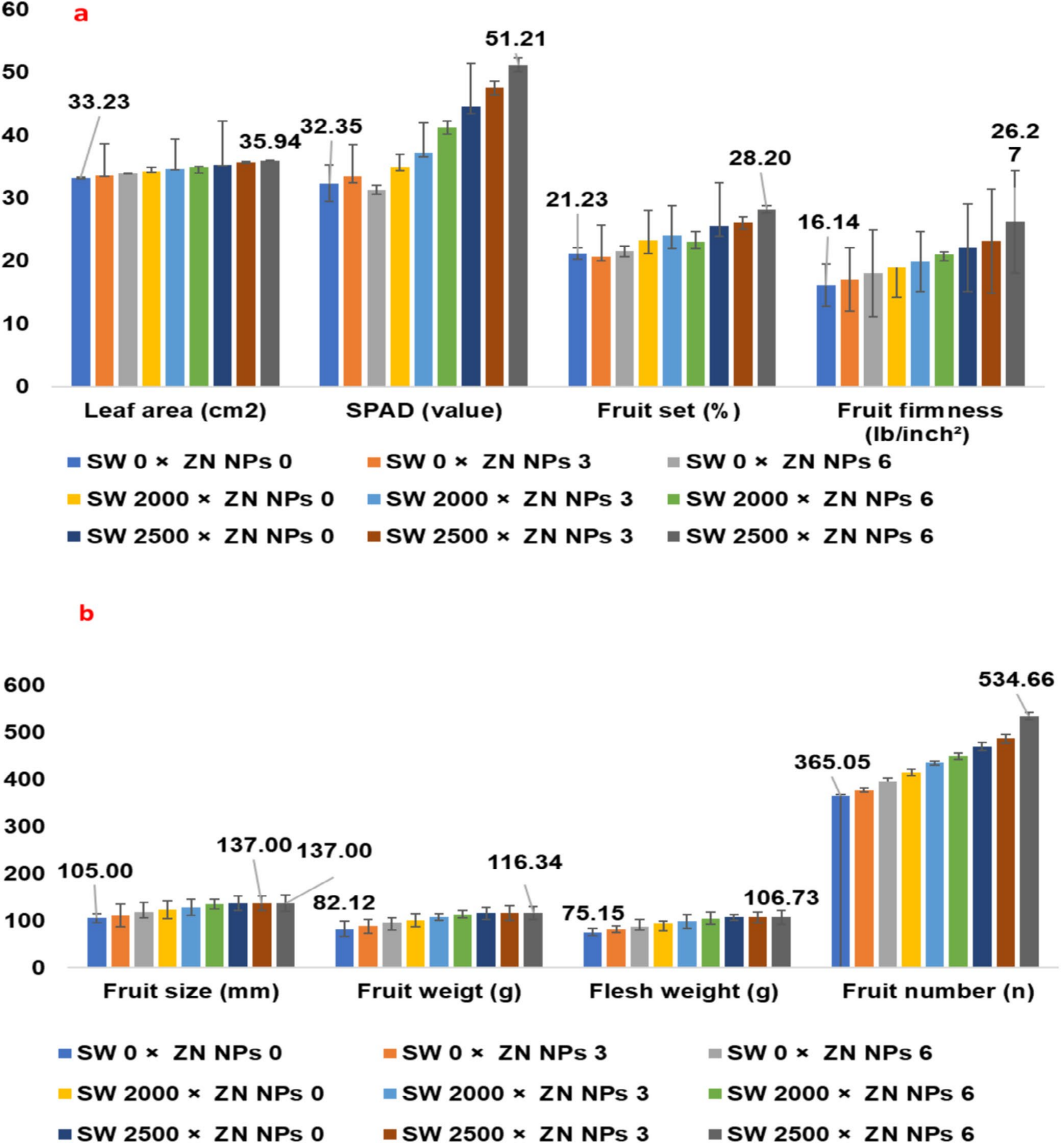
The interaction effect of foliar application of seaweed extract (*Spirulina platensis*) (SW) and nano‐zinc levels: (a) Leaf area, SPAD value, fruit set, fruit firmness, and (b) fruit size (diameter), fruit weight, flesh weight, seed weight, and fruit number of peach trees; Tukey's test, α = 0.05, *n* = 30.

The interaction of seaweed (*Spirulina platensis*) and nano‐zinc was significant for leaf area. The control treatment exhibited the lowest value of leaf area (33.23 ± 0.12 cm^2^), whereas the highest was observed at 2500 ppm seaweed (*Spirulina platensis*) and 6 mL L^−1^ Zn NPs (35.94 ± 0.07 cm^2^) (Figure 2a). The minimum SPAD was observed in control plants (32.35 ± 2.90), increasing to 51.21 ± 1.12 at 2500 ppm *Spirulina platensis* and 6 mL L^−1^ Zn NPs. Furthermore, a notable improvement (41.19 ± 1.11) was evident in response to 2000 ppm seaweed (*Spirulina platensis*) and 6 mL L^−1^ Zn NPs (Figure 2a). Fruit set percentage demonstrated positive responses to combined levels of *Spirulina platensis* extract and Nano‐Zinc. The control plants yielded the least fruit formation (21.23% ± 0.90%), while the maximum fruit set (28.20% ± 0.61%) was observed in trees which were treated with 2500 ppm seaweed (*Spirulina platensis*) and 6 mL L^−1^ Zn NPs (Figure 2a).

Fruit diameter (mm) showed steady augmentation in response to all treatments. The smallest fruits (105.0 ± 20 mm) were measured from control trees, while the biggest fruits (137 ± 3 mm) were obtained under application of 2500 ppm *Spirulina platensis* extract + 3 or 6 mL L^−1^ Zn NPs (Figure 2b). The control plants recorded a minimum fruit weight (82.12 ± 2.09 g), whereas the highest fruit weight (116.34 ± 1.68 g) was noted at 2500 ppm seaweed extract (*Spirulina platensis*) and 6 mL L^−1^ Zn NPs (Figure 2b). Furthermore, flesh weight (g) exhibited a similar trend to fruit weight, increasing from 75.15 ± 1.65 g in the control to 106.73 ± 1.14 g in the trees treated with the highest level of treatments (2500 ppm *Spirulina platensis* + 6 mL L^−1^ Zn NPs) (Figure 2b). Fruit number per tree increased with application of seaweed extract (*Spirulina platensis*) and Zn NPs levels, from 365.05 ± 3.37 in the control to 534.66 ± 19.53 at plants treated with 2500 ppm *Spirulina platensis* extract and 6 mL L^−1^ Zn NPs (Figure 2b).

**FIGURE 3:**
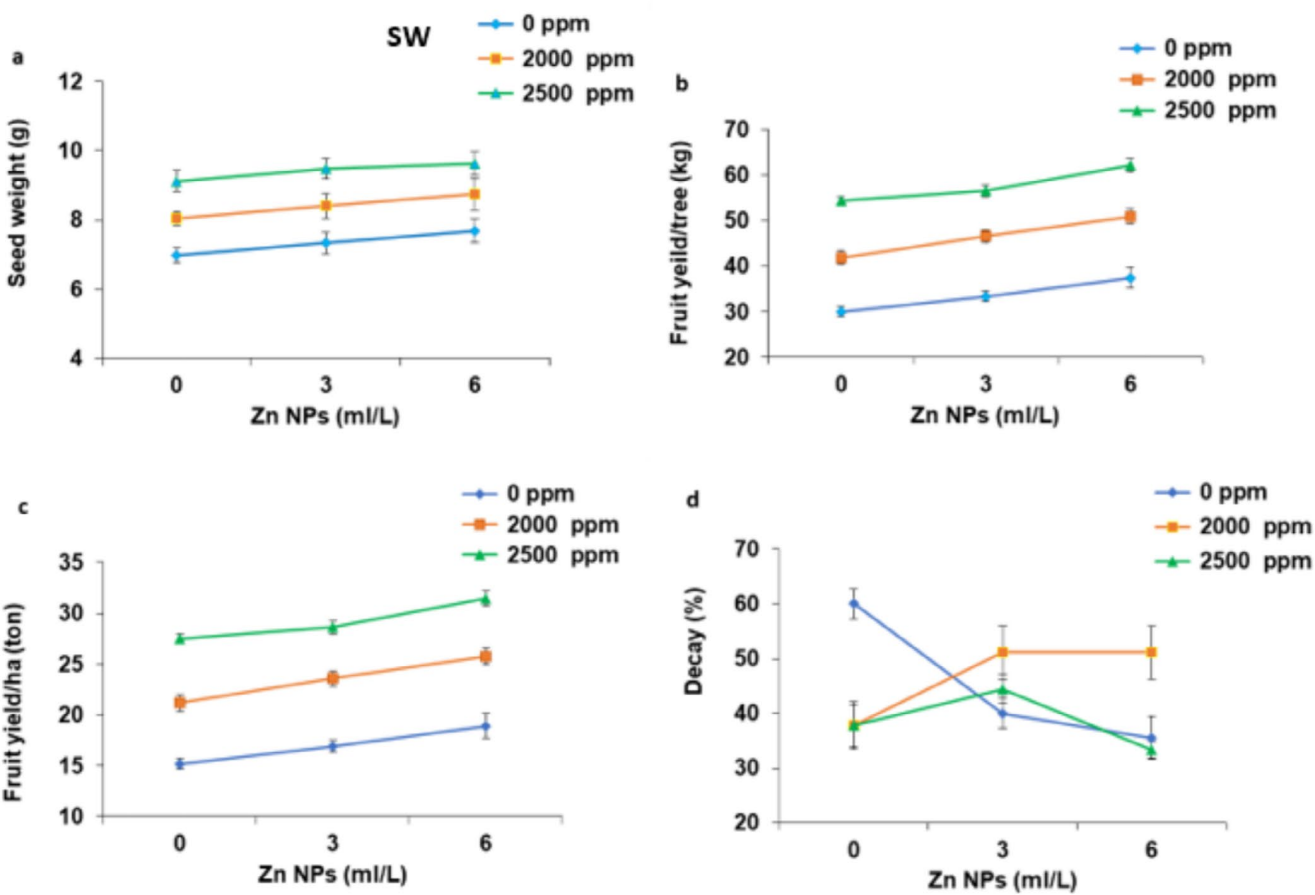
The interaction effect of foliar application of seaweed extract (*Spirulina platensis*) (SW) and nano‐zinc levels on fruit yield per tree (a), fruit yield ha^−1^ (b), fruit firmness (c), and fruit decay (d) of peach; Tukey's test, *α* = 0.05, *n* = 30.

The lowest value of seed weight (6.97 ± 0.12 g) obtained under no fertilizers application (control) and the peak of seed weight (9.61 ± 0.34 g) in response to 2500 ppm *Spirulina platensis* + 6 mL L^−1^ Zn NPs (Figure 3a). The lowest fruit yield per tree (29.98 ± 0.94 kg) was obtained from the control trees and the highest yield (62.20 ± 2.29 kg) from those which were treated with 2500 ppm seaweed extract (*Spirulina platensis*) + 6 mg L^−1^ Nano‐Zinc (Figure 3b). Also, fruit yield per hectare increased from 15.17 ± 0.48 ton ha^−1^ in the control (minimum) to 31.47 ± 1.16 ton ha^−1^ for trees under treatment with 2500 ppm seaweed extract (*Spirulina platensis*) and 6 mL L^−1^ Zn NPs (Figure 3c). Seaweed notably affected the fruits quality characteristics including TSS and fruit firmness (*p* ≤ 0.01) and total acidity at the 0.05 level, while Zn‐NPs influenced meaningfully TSS and fruit firmness (*p* ≤ 0.01) and total acidity at the 0.05 level. Fruit decay was influenced by combined treatments of seaweed extract (*Spirulina platensis*) and Nano‐Zinc concentration at the 0.01 level. Fruit firmness was improved (non‐significantly) from 16.14 ± 0.30 lb in.^−2^ in the control fruits to 26.27 ± 1.90 lb in.^−2^ for fruits obtained from trees under application of 2500 ppm *Spirulina platensis* extract + 6 mL L^−1^ Zn NPs (Figure 3d). Finally, decay percentage decreased inversely with treatment levels. The highest decay of 60.00% ± 2.67% occurred in the control fruits, dropping significantly to 33.33% ± 1.55% in fruits gained from trees which were treated with 2500 ppm *Spirulina platensis* extract + 6 mL L^−1^ Zn NPs, indicative of improved post‐harvest resistance (Figure 3d).

**FIGURE 4:**
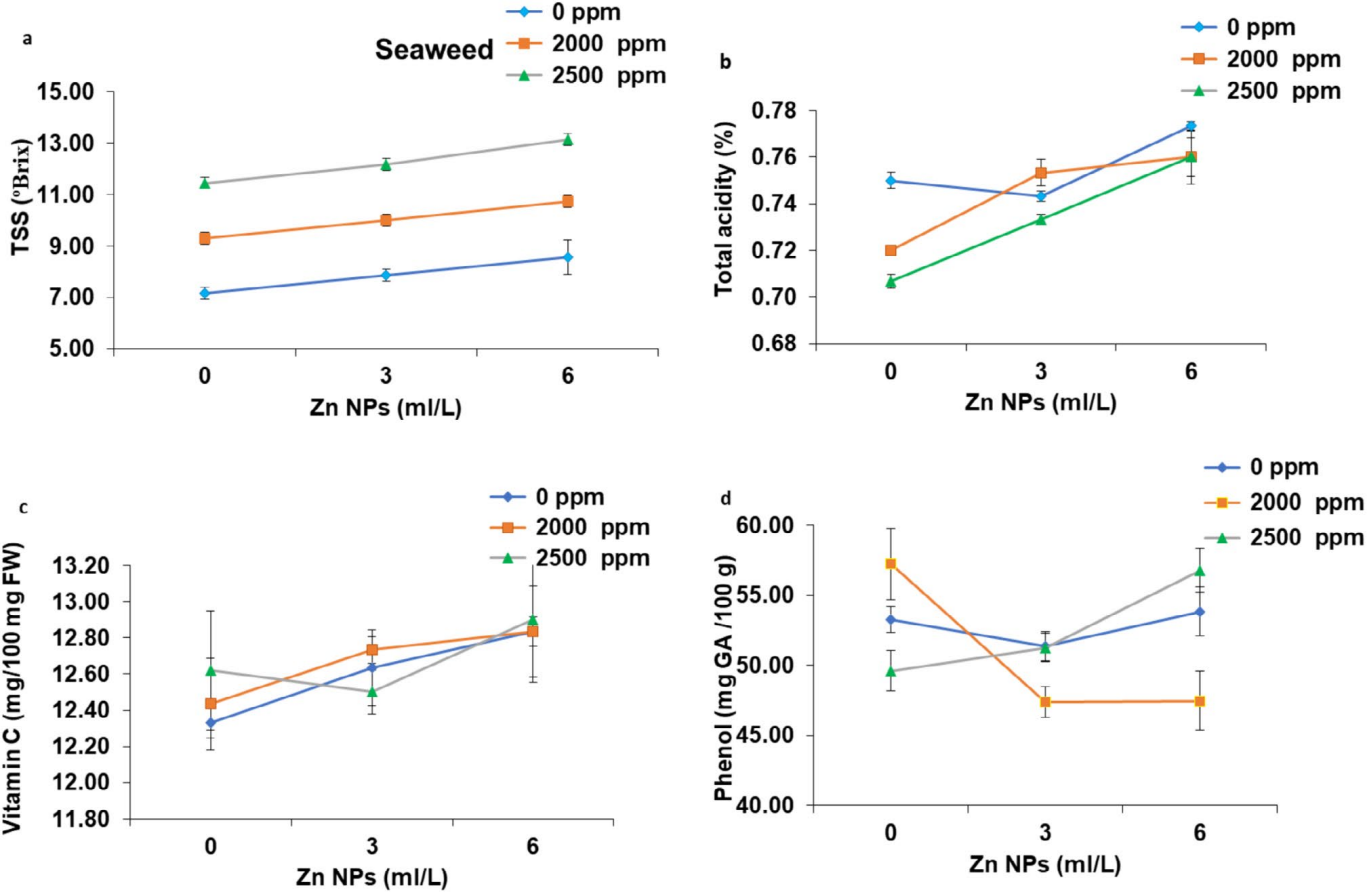
The interaction effect of foliar application of seaweed extract (*Spirulina platensis*) and nano‐Zinc levels on fruit TSS (a), total acidity of peach fruits (b), vitamin C content (c), and phenol content of fruit (d); Tukey's test, *α* = 0.05, *n* = 30.

Total soluble solids content, a key quality indicator for fruits rose from 7.16 ± 0.22 (°Brix) in the control fruits to 13.15 ± 0.67 (°Brix) (highest value) in the fruits obtained under effect of foliar application of seaweed extract (*Spirulina platensis*) and Nano‐Zinc (2500 ppm + 6 mL L^−1^), suggesting sweeter and more flavorful fruits (Figure 4a). Total acidity (%) showed minimal variation, but overall slight declines (non‐significantly) under application of *Spirulina platensis* and Zn NPs, from 0.75% ± 0.001% in the control fruits to 0.71% ± 0.001% under application of 2500 ppm seaweed (*Spirulina platensis*) and 0 mL L^−1^ Zn NPs (Figure 4b). Vitamin C content (mg/100 mg FW) exhibited moderate (non‐notably) increases under effect of *Spirulina platensis* and Nano‐Zinc levels, ranging from 12.53 ± 0.25 mL/100 mL in the control peaches to 12.90 ± 0.35 mL/100 mL with application of 2500 ppm seaweed extract (*Spirulina platensis*) + 6 mL L^−1^ Zn NPs (Figure 4c). Finally, total phenolic content was increased substantially with application of *Spirulina platensis* and Zn NPs, from 53.27 ± 0.90 mg GAE·100 g^−1^ in the control fruits to 57.23 mg GAE·100 g^−1^ at 2000 ppm *Spirulina platensis* and 0 mL L^−1^ Zn NPs (peak value) with foliar application of 2500 ppm seaweed extract (*Spirulina platensis*) + 6 mL L^−1^ Zn NPs treatment implying greater antioxidant potential and health benefits (Figure 4d).

### 3.1 Principal Component Analysis (PCA)

**FIGURE 5:**
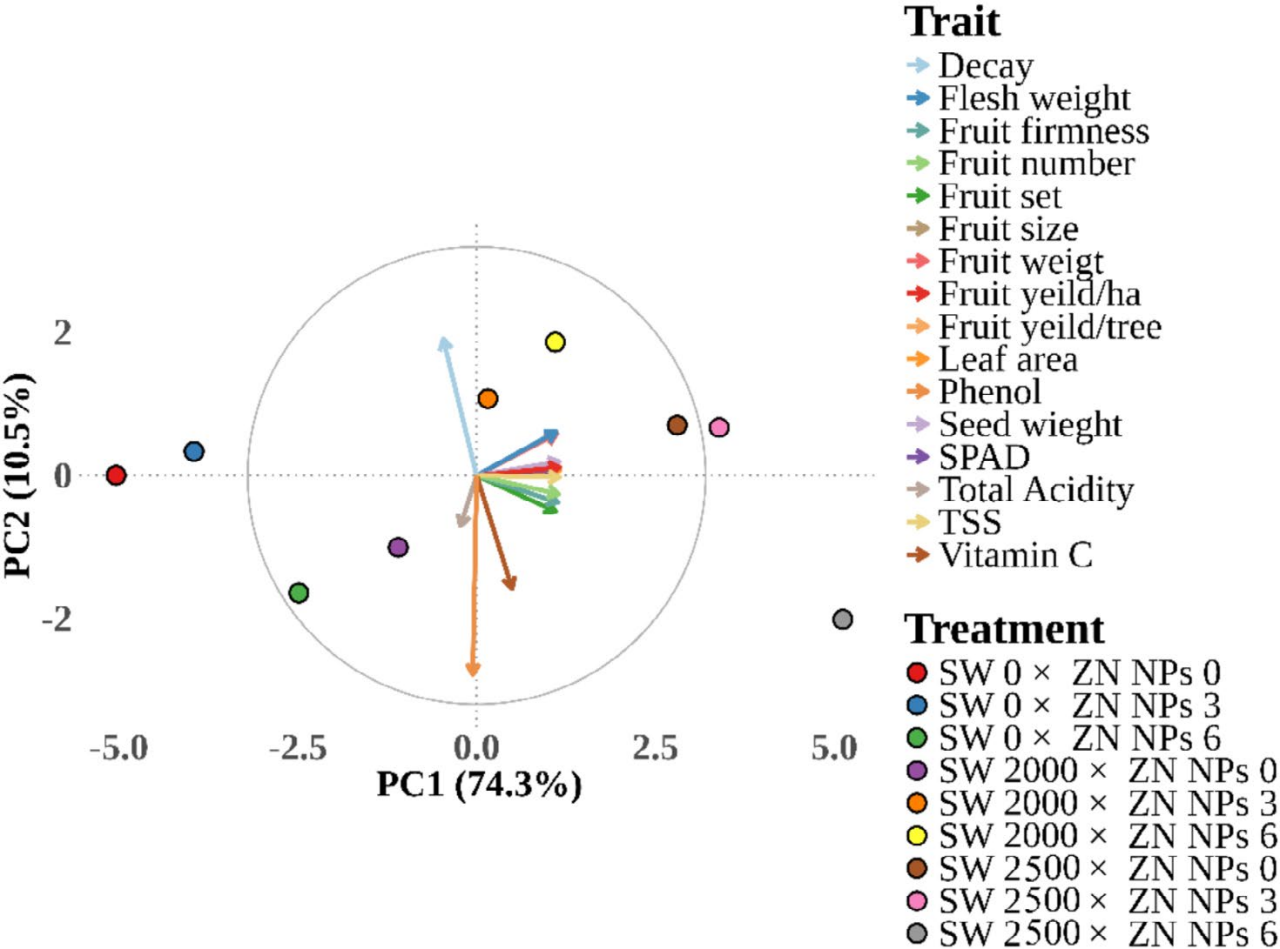
Relations between different seaweed extract (*Spirulina platensis*) (SW) × Zn NPs treatments and studied variables of peach based on the biplot graph for PC1 and PC2.

PCA was performed to elucidate the multivariate relationships among the measured traits and to visualize the distribution of the treatment combinations (Figure 5). The first two components explained 84.8% of the total variance (PC1 74.3% and PC2 10.5%). All vegetative, reproductive, and yield‐related traits loaded positively and strongly on PC1 (Table 2). In contrast, fruit decay, total acidity, and phenol content exhibited weak negative loadings on PC1. PC2, explaining an additional 10.5% of variance, showed post‐harvest and biochemical quality contrasts. Decay loaded positively and most strongly on PC2 (0.477).

**TABLE 2:** The shares of treatments in the PC1 and PC2.

Variable	PC1	PC2	Variable	PC1	PC2
Leaf area	0.289	0.018	Seed weight	0.289	0.049
SPAD	0.276	0.021	Fruit yield per tree	0.290	0.028
Fruit set	0.276	−0.125	Fruit yield ha^−1^	0.290	0.028
Fruit firmness	0.284	−0.094	Decay	−0.117	0.477
Fruit diameter	0.279	0.151	TSS	0.289	−0.006
Fruit weight	0.280	0.146	Total Acidity	−0.057	−0.176
Flesh weight	0.279	0.153	Vitamin C	0.124	−0.394
Fruit number	0.287	−0.065	Phenol	−0.013	−0.696
Eigenvalue	11.890	1.681	Eigenvalue	11.890	1.681
Proportion	0.743	0.105	Proportion	0.743	0.105
Cumulative	0.743	0.848	Cumulative	0.743	0.848

Treatments with 0 ppm seaweed were extract (*Spirulina platensis*) characterized by low yield, small fruit diameter, high decay, and minimal quality enhancement, particularly the control treatment (0 × 0) and 0 × 3 mL L^−1^ Zn NPs. The treatments containing 2000 ppm *Spirulina platensis* showed moderate improvements in yield and quality. The combined treatments of nano zinc with 2500 ppm *Spirulina platensis* closely aligned with fruit yield, number, weight, diameter, firmness, and TSS, while they showed an opposite trend with fruit decay.

The treatment of 0 ppm seaweed extract (*Spirulina platensis*) × 3 mL L^−1^ Zn NPs showed a minor relation with fruit set and SPAD, while it remains strongly tied to fruit decay, such that it slightly improved chlorophyll and fruit initiation, while fruit yield and size remain low. The treatment of 6 mL L^−1^ Zn NPs (without *Spirulina platensis* extract) highly correlated to phenol content and moderately associated with vitamin C content, suggesting the highest phenolic content among low‐seaweed (*Spirulina platensis*) treatments. However, this treatment gained poor yield, small fruit size and weight, low firmness, and high decay, indicating that 6 mL L^−1^ Zn NPs alone enhance antioxidant compounds but not peach growth and fruit yield. Seaweed extract (*Spirulina platensis*) at 2000 ppm correlated to high phenol and vitamin C contents with intermediate growth and yield, but decay is still elevated. The application of 2000 ppm seaweed extract (*Spirulina platensis*) × 3 mL L^−1^ Zn NPs represented moderate enhancement in fruit quality and yield, with reduced fruit decay compared to control. Seaweed extract (*Spirulina platensis*) at 2000 ppm × 6 mL L^−1^ Zn NPs closely associated with fruit yield, size, and weight, and in addition, SPAD value and leaf area. Furthermore, it slightly correlated to reduced fruit decay, thereby significantly improving fruit yield and vegetative growth, but it is less optimal in post‐harvest stability compared to higher *Spirulina platensis* doses (2500 ppm). The treatment of 2500 ppm seaweed extract (*Spirulina platensis*) × 3 mL L^−1^ Zn NPs associated with near‐maximal productivity and fruit quality, and led to excellent balance across fruit qualitative traits. Finally, 2500 ppm seaweed extract (*Spirulina platensis*) × 6 mL L^−1^ Zn NPs was highly associated with fruit yield, number, weight, size, firmness, and TSS, furthermore with SPAD, and leaf area. However, it represented the lowest association with fruit decay, as well as moderate linkage to vitamin C and phenol content. This treatment maximizes nearly all desirable traits including largest fruits, highest yield per tree and hectare, greatest fruit number, firmness, sweetness (TSS), leaf chlorophyll content, and leaf area, with minimal post‐harvest decay. Thus, the 2500 ppm seaweed extract (*Spirulina platensis*) + 6 mL L^−1^ Zn NPs treatment is unambiguously optimal, achieving the highest productivity, fruit diameter, weight, firmness, sweetness, and post‐harvest stability.

### 3.2 The Results of Pearson's Correlation

**FIGURE 6:**
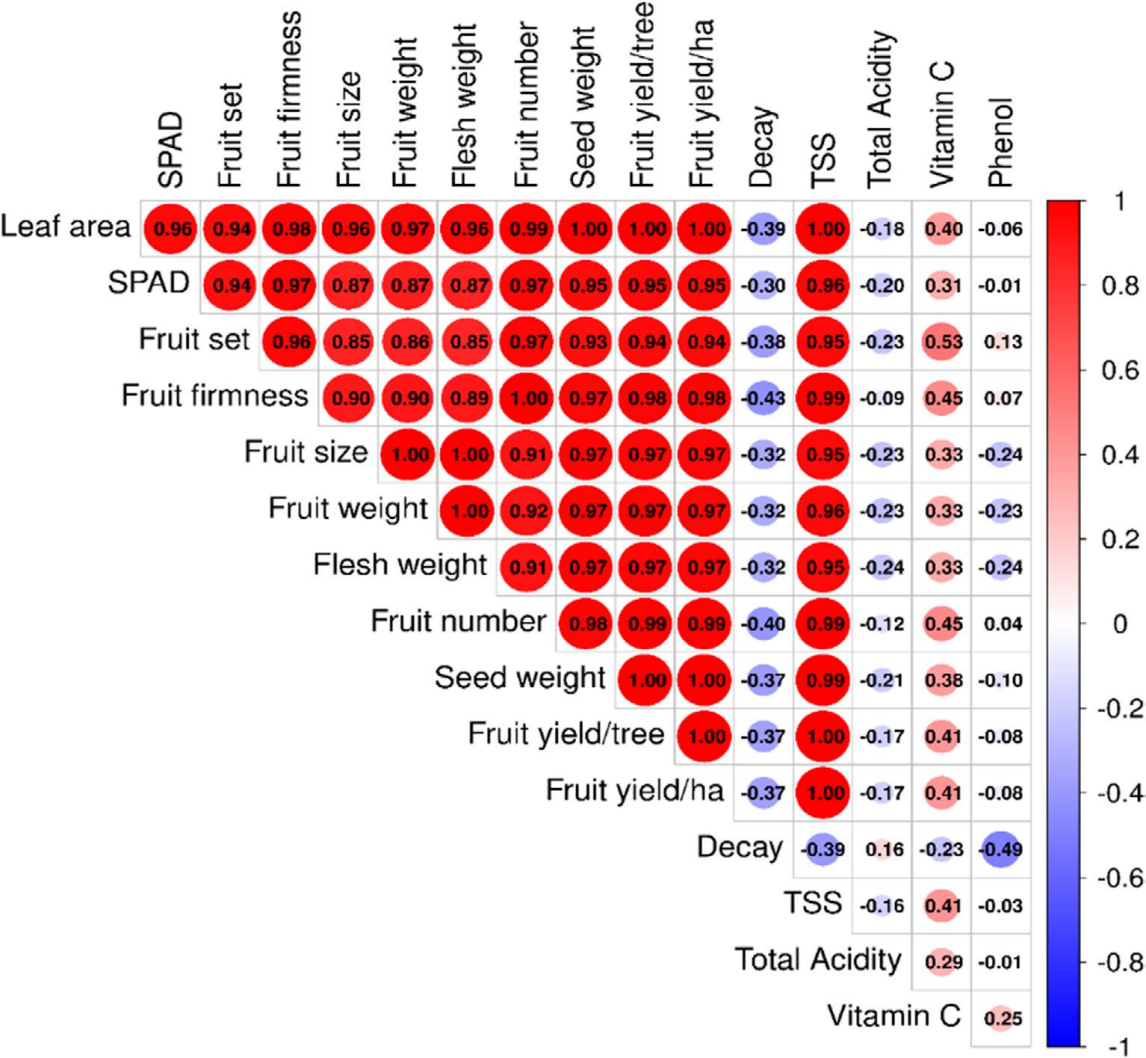
The correlation graph between different studied peach variables under different seaweed extract (*Spirulina platensis*) × Zn NPs treatments.

Overall, the highly positive correlations were observed between physical, morpho‐physiological and yield variables, mostly above 0.87** (Figure 6). Fruit yield exhibited extreme positive associations with leaf area (*r* = 0.992**), TSS (*r* = 0.990**), seed weight (*r* = 0.980**), fruit firmness (*r* = 0.980**), fruit number (*r* = 0.970**), fruit size (*r* = 0.970**), fruit weight (*r* = 0.998**), flesh weight (*r* = 0.999**), fruit set (*r* = 0.940**), and SPAD value (*r* = 0.940**). Similarly, leaf area was very strongly correlated with fruit number (*r* = 0.990**), TSS (*r* = 0.990**), fruit firmness (*r* = 0.980**), seed weight (*r* = 0.980**), fruit size (*r* = 0.970**), fruit weight (*r* = 0.970**), flesh weight (*r* = 0.960**), SPAD (*r* = 0.940**), and fruit set (r = 0.940**). SPAD value showed very strong positive correlations with fruit number (*r* = 0.970**), fruit firmness (*r* = 0.940**), fruit size (*r* = 0.940**), fruit weight (*r* = 0.940**), fruit set (*r* = 0.940**), TSS (*r* = 0.940**), seed weight (*r* = 0.930**), and flesh weight (*r* = 0.870**). Fruit decay had moderate per weak low negative correlations with fruit yield per tree and per hectare (*r* = −0.400), leaf area (*r* = −0.390), TSS (*r* = −0.390), fruit number (*r* = −0.370), fruit weight (*r* = −0.370), flesh weight (*r* = −0.370), seed weight (*r* = −0.370), fruit firmness (*r* = −0.320), fruit size (*r* = −0.320), SPAD value (*r* = −0.300), and fruit set (*r* = −0.300). Vitamin C and phenol were moderately correlated (positively) with each other (*r* = 0.410) and with seed weight (*r* = 0.410), fruit set (*r* = 0.450), fruit firmness (*r* = 0.450), fruit size (*r* = 0.450), and TSS (*r* = 0.410).

### 3.3 Regression

**FIGURE 7:**
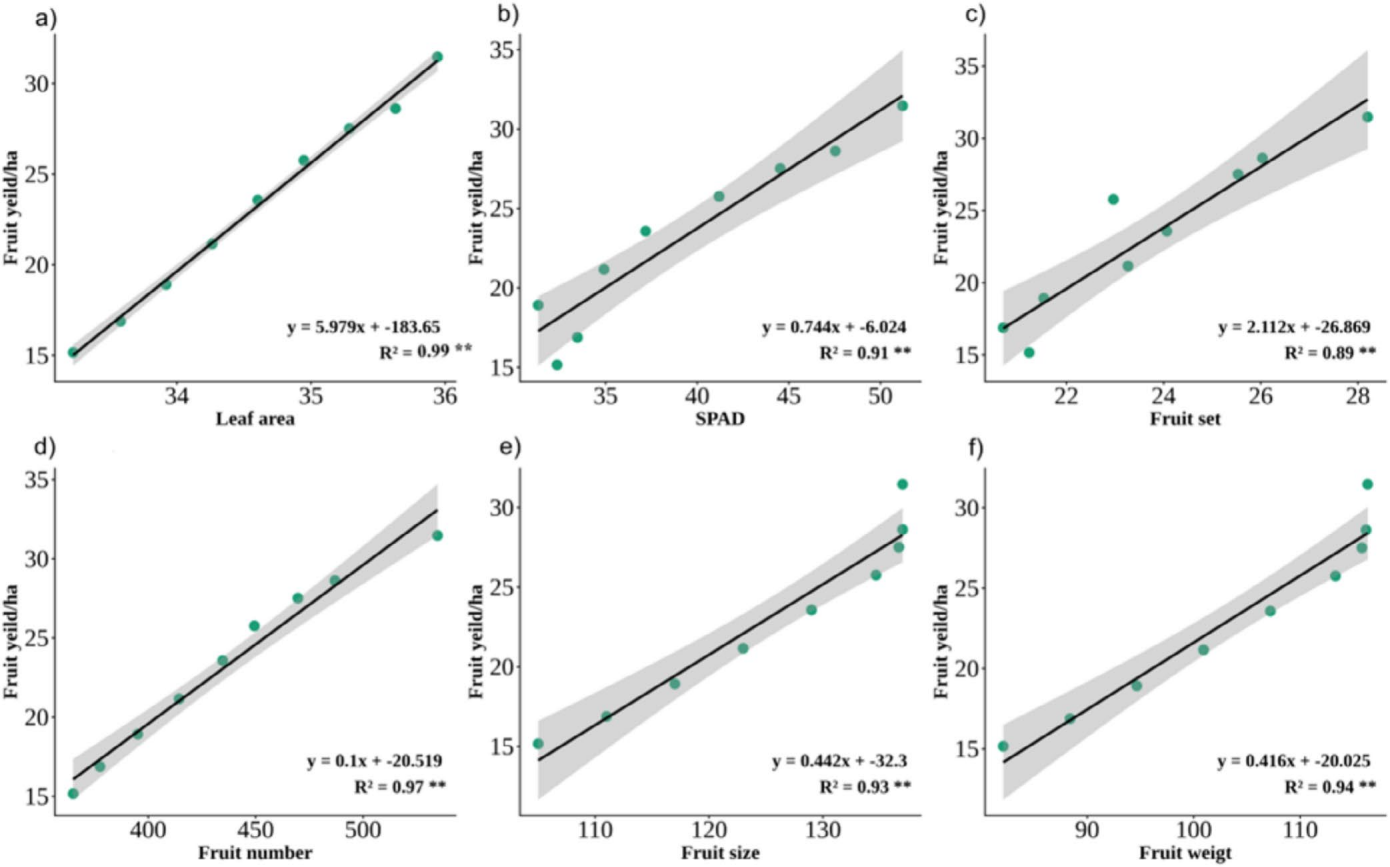
The regression relations between fruit yield as dependent variable and predictors: (a) Leaf area, (b) SPAD, (c) fruit set, (d) fruit number, (e) fruit diameter, and (f) fruit weight in peach under application of different levels of seaweed extract (*Spirulina platensis*) and nano zinc.

Stepwise regression analysis revealed the significant correlations between Fruit yield (dependent variable) and predictors including leaf area, SPAD value, fruit set, fruit number, fruit size, and fruit weight (Table 3). Fruit yield exhibited a very strong relationship with leaf area (*y* = 5.979*x*–183.65, *R* ^2^ = 0.99**), indicating that increases in leaf area led to higher yield (Figure 7a). Similarly, significant positive relationships were observed between fruit yield and SPAD values (*y* = 0.744*x*–6.024, *R* ^2^ = 0.91**) (Figure 7b), fruit set (*y* = 2.112*x*–26.869, *R* ^2^ = 0.89**) (Figure 7c), fruit number (*y* = 0.1*x* + 20.519, *R* ^2^ = 0.97**) (Figure 7d), fruit size (*y* = 0.442*x*–32.3, *R* ^2^ = 0.93**) (Figure 6e), and fruit weight (*y* = 0.416*x*–20.025, *R* ^2^ = 0.94**) (Figure 7f). Leaf area and fruit number had the strongest associations with fruit yield and were the most influential determinants of fruit yield. The high *R* ^2^ values across all regressions confirm that variations in the studied physiological and yield components explain the majority of the variation in fruit yield. Thus, these variables are emphasized as reliable indicators for predicting productivity.

**TABLE 3:** The results of stepwise regression for fruit yield^a^ and predictors.

Model	Intercept	Slope	*p*	Sig.	*R* ^2^	Adjusted_*R* ^2^
Fruit yield ha^−1^ vs. Leaf area	−183.65	5.979	1.78E‐09	**	0.9955	0.9949
Fruit yield ha^−1^ vs. SPAD	−6.024	0.744	6.61E‐05	**	0.9099	0.8971
Fruit yield ha^−1^ vs. Fruit set	−26.869	2.112	1.50E‐04	**	0.8865	0.8702
Fruit yield ha^−1^ vs. Fruit diameter	−32.3	0.442	2.18E‐05	**	0.9343	0.9249
Fruit yield ha^−1^ vs. Fruit weight	−20.025	0.416	1.50E‐05	**	0.9409	0.9324
Fruit yield ha^−1^ vs. Fruit number	−20.519	0.1	8.18E‐07	**	0.9741	0.9704

^a^ Dependent variable.

** Significant at the 0.01 level.

## 4 Discussion

Application of seaweed extract (*Spirulina platensis*) and/or nano‐zinc promoted fruit development and enhanced fruit quality. However, application of 2500 ppm +6 mg L^−1^ Zn NPs had the highest influence on fruit production and fruit quality. This treatment led to the highest improvements in leaf area, SPAD value, fruit set, fruit number, fruit diameter, fruit weight, flesh weight, seed weight, fruit yield, fruit firmness, and TSS content, while it highly reduced fruit decay. This growth development can be ascribed to the advantageous influence of seaweed extract (*Spirulina platensis*), which contains essential macro and microelements. These nutrients accomplish the plant's needs for photosynthesis, respiration, cell division, and elongation. Also, microelements are essential for protein synthesis, chlorophyll synthesis, IAA production, and cell division, resulting in the regulation of photosynthesis, protein synthesis, and the uptake of nutrients and water, ultimately endorsing boosted plant growth (Ujjwal et al. 2025). Furthermore, the availability of Zinc as a mandatory trace element activator for numerous plant enzymes and directly involved in the biosynthesis of growth hormones, resulting in improved cell division and cell elongation (Mahmoud et al. 2020). Foliar application of nano‐fertilizers combined with natural biostimulants has been shown to enhance growth and fruit quality in horticultural crops. Hamzah and Ibrahim (2024) reported that nano and organic fertilizers improved leaf area, chlorophyll content, and nutrient accumulation in sour orange. Similarly, in the present study, foliar application of nano‐chelated zinc with *Spirulina platensis* seaweed extracts increased leaf area, SPAD values, fruit set, yield, and fruit quality in peach, highlighting the synergistic potential of eco‐friendly fertilization strategies. This confirms that combining nanotechnology with natural biostimulants can be an effective approach to improve morpho‐physiological traits and post‐harvest fruit quality in fruit trees.

A review study reported that seaweed fertilizer (*Spirulina platensis*) overall, led to significant average increase in crop yield by 15.17%, tuber yield by 21.19%, sugar acid ratio (38.32%), vitamin C (18.07%), starch (19.65%), and protein (11.45%) (Pei et al. 2024). Application of Spirulina platensis 6 mL L^−1^ + Zinc Sulphate 600 mg L^−1^ led to maximal plant height (28.47%) rootstock girth (32.39%) and scion girth (20.08%) No. of shoots per branch (3.74%), No. of leaves per plant (27.91%), Leaf length (27.89%), Leaf width (13.65%), and Leaf area (24.40%) control. This improvement can be attributed to the beneficial impact of *Spirulina platensis* seaweed extract, which contains essential macro elements (N, P, K and Mg) and microelements (Fe, Zn, Cu and Mn). These elements fulfill the plant's mineral needs for processes such as photosynthesis, respiration, cell division and elongation. The improved growth of plants may be attributed to the increased availability of microelements such as Fe, Zn, Cu and Mn. These microelements are essential for protein synthesis, chlorophyll formation, as well as the production of the growth hormone IAA and cell division. These processes collectively contribute to the regulation of cell respiration, photosynthesis, protein synthesis and the uptake of nutrients and water, ultimately promoting enhanced plant growth. Recently, reports confirmed the important of bioactive substances derived from seaweed in Agri‐Food productions (Deolu‐Ajayi et al. 2022; Lomartire and Gonçalves 2022; Pradhan et al. 2021; Zhang et al. 2022). These bioactive materials enhance stress resistance and efficiency of water and fertilizers (Deolu‐Ajayi et al. 2022; EL Boukhari et al. 2020; Gatti et al. 2025; Supriya et al. 2024). In additions, beside plant growth hormones, seaweed supply nutrients, amino acid, and protein for plant growth and development (Ali et al. 2022; Kumawat and Kumawat 2023; Ouala et al. 2025; Rabhi et al. 2025).

In past studies, seaweed has proven significant improvements on growth, yield, quality, storage and stress tolerance in a variety of fruit crops (Rajendra et al. 2024). In grapevine berries, foliar spraying with NPs‐ZnO definitely alters yield, and bioactive compounds. Also, 50–75 mg L^−1^ of NPs‐ZnO increased crop yield, antioxidants, and Zn concentration (GuillÉN‐EnrÍQuez et al. 2023). Seaweed extract at 3000 ppm has improved significantly fruit length, diameter, weight, and total yield of kiwifruit, fruit soluble solids contents and total sugars. Furthermore, the application of 2000 ppm seaweed exhibited the lowest physiological weight loss and the highest ascorbic acid content (Rana et al. 2023). In ‘Golden Delicious’ apple, by increasing the concentration of seaweed extracts, the concentration of N, Fe, and P, chlorophyll, fruit diameter, and yield has increased (Mousavi et al. 2024). In orchards of far north Queensland, Australia, the use of seaweed extract meaningfully enhanced avocado yield by 38%, fruit firmness by 22%, and number of fruits per tree up to 42% (Arioli et al. 2024). *Pyrus communis* ‘William's’ treated with seaweed recorded an average increase of diameter (6%), weight (20%) and seed number (40%) (Colavita et al. 2011). The seaweed knowingly augmented the photosynthetic rate, chlorophyll content and Rubisco enzyme activity in ‘Fuji’ apple leaves. It also, has improved the quality of ‘Fuji’ apples fruit in terms of fruit weight, soluble solid content (SSC), soluble sugar content, sugar–acid ratio, vitamin C (VC) content, and free amino acid ‐treated apples increased by 10.74%, 12.16%, 21.96%, 45.12%, 56.12%, 47.96%, respectively (Yang et al. 2023). Exogenous seaweed extract (*Nizamuddinia zanardinii*) pointedly has amended total carbohydrate content, total soluble solids (TSS), pH, and fruit yield of tomato (Jalali et al. 2022). The use of green algae and amino acids were tested as biostimulants on three hot pepper cultivars. A large variability was observed between the effects of the two biostimulants on the cultivars. Green algae‐treated ‘Somborka’ and ‘Habanero Red Caribbean’ has increased by 10% in seeds dry matter compared to control treatment (Zamljen et al. 2021). The 3.76 g L^−1^ concentration of Ulva flexuosa extract maximized the improvement of antioxidant capacity, ascorbic acid content and TSS during days of storage of Cucumis (Rezaei et al. 2019). In Navelina orange, 0.15% seaweed extract has increased the yield by 8% whereas 0.30% it increased the yield by 15% (Fornes et al. 2002). The mentioned examples confirmed the present results which we observed in peach fruits.

Zinc (Zn), a crucial micronutrient in plant fertilization programs, is involved in plant physiological processes, such as photosynthesis, carbohydrate and protein metabolism, and growth regulation for fortifying the crop yield and quality (Dang et al. 2024; Hamzah et al. 2022; Li et al. 2022; Liu et al. 2025; Meriño‐Gergichevich et al. 2021; Umair Hassan et al. 2020; Veena and Puthur 2022). Zinc is well‐known as a vital activator of several enzymes in plants. Besides, it is directly involved in the biosynthesis of growth substances such as auxin, which produces more plant cells, thus increasing the dry matter (Mahmoud et al. 2020). Zinc deficiency causes disruption of plant metabolism, growth restriction, and yield reduction (Ahmed, Deng, et al. 2024; Khan et al. 2022; Sethi et al. 2025; Tayyiba et al. 2021, Nandal and Solanki 2021). Zn is involved in tryptophan biosynthesis, a necessary amino acid for biosynthesis of IAA, which controls cell expansion and elongation (Dang et al. 2024; Umair Hassan et al. 2020). Zinc contributed to plant homeostasis and regulatory mechanisms in horticulture crops (Manzoor et al. 2025; Song and Kim 2020), such as cell membrane rigidity, chloroplast development, and the release of endogenous hormones (Vadlamudi et al. 2020). Furthermore, it is a main cofactor of ligases, hydrolases, isomerases, and transferases enzymes, which are in role to cellular metabolism adjustments (Cakmak 2000; García‐López et al. 2019). Some studies have cleared that Zn deficiency impairs zinc‐dependent enzymatic and metabolic systems and leads to physiological stress (Coşkun et al. 2025; Hamzah et al. 2022).

In crops, application of ZnO NPs (0.12 g pot^−1^) significantly increased chlorophyll B contents by 24.6%, plant height by 34.6%, and wheat grain yield by 42.2%, respectively (Adil et al. 2022). Also, application of 150 ppm ZnO‐NPs meaningfully augmented the wheat grains number by 12.5%, grain weight (12.4%), total yield (25.5%), and zinc contents (58.6%) compared to the control (Raza et al. 2025). Furthermore, the foliar application of ZnO‐NPs increased the plant height by 17.9%, dry matter by 15.1% and leaf area index by 69.4%, grain yield by 16.9% in wheat plants (Omkar et al. 2023). Over all, in present study, PCA demonstrated that the combination of 2500 ppm seaweed extract (*Spirulina platensis*) and 6 mL L^−1^ Zn NPs emerges as the optimal treatment, maximizing yield and growth traits while maintaining low decay and high biochemical quality. The variation in fruit decay between different concentrations of Spirulina extract may be attributed to dose‐dependent physiological responses. While moderate concentrations (e.g., 2000 ppm) can enhance antioxidant activity and strengthen cell wall integrity, higher concentrations (e.g., 2500 ppm) may lead to excessive metabolic activity, increased tissue softness, or imbalanced nutrient uptake. additionally, high concentrations of biostimulants may alter microbial interactions on the fruit surface, potentially influencing decay incidence. Therefore, although higher doses improved several growth and quality traits, their effects on post‐harvest stability may not always follow a linear trend. Principal Component Analysis (PCA) has revealed the potential of S*pirulina platensis* to enhance the phytochemical profile of apricot fruits (Gatti et al. 2025).

## 5 Conclusion

The results of this study demonstrated that foliar application of *Spirulina platensis* extract, nano‐chelated zinc, and especially their combined use significantly improved morphophysiological traits, fruit yield, post‐harvest quality, and biochemical characteristics of peach fruits. Among all treatments, the integration of 2500 ppm *Spirulina platensis* extract + 6 mL L^−1^ nano‐zinc proved to be the most effective, resulting in the highest values of leaf area, SPAD index, fruit set, fruit diameter and weight, fruit yield per tree and hectare, firmness, and TSS, while minimizing decay percentage during storage. Correlation, PCA, and regression analyses confirmed that improvement in physiological characteristics such as leaf area and SPAD strongly contributed to higher productivity. Based on these findings, combined foliar application of seaweed and nano‐zinc is recommended as an eco‐friendly and efficient approach for enhancing peach production and fruit marketability under similar environmental conditions. Certain limitations of this study should be noted. Data were collected during a single growing season, which may limit the generalizability of the findings; however, peach trees were exposed to different ds, which can be considered as different environments. We suggest multi‐year, multi‐varietal experiments in future work.

## Author Contributions

**Ahmed Isam Dawood:** investigation, visualization, project administration. **Lina saadoun Abdul majeed:** visualization, formal analysis, project administration, resources, supervision. **Saad Abdulmajeed Waheeb:** writing – original draft, data curation, methodology, validation. **Heidar Meftahizadeh:** writing – review and editing, data curation, supervision, software.

## Funding

The authors have nothing to report.

## Ethics Statement

This study is not a clinical trial and no human participants are involved in this research.

## Consent

The authors declare their consent to the publication of this article.

## Conflicts of Interest

The authors declare no conflicts of interest.

## Data Availability

All data generated during this study are included in this article.
